# Pollen- and Seed-Mediated Transgene Flow in Commercial Cotton Seed Production Fields

**DOI:** 10.1371/journal.pone.0014128

**Published:** 2010-11-30

**Authors:** Shannon Heuberger, Christa Ellers-Kirk, Bruce E. Tabashnik, Yves Carrière

**Affiliations:** Department of Entomology, University of Arizona, Tucson, Arizona, United States of America; Purdue University, United States of America

## Abstract

**Background:**

Characterizing the spatial patterns of gene flow from transgenic crops is challenging, making it difficult to design containment strategies for markets that regulate the adventitious presence of transgenes. Insecticidal *Bacillus thuringiensis* (Bt) cotton is planted on millions of hectares annually and is a potential source of transgene flow.

**Methodology/Principal Findings:**

Here we monitored 15 non-Bt cotton (*Gossypium hirsutum*, L.) seed production fields (some transgenic for herbicide resistance, some not) for gene flow of the Bt cotton *cry1Ac* transgene. We investigated seed-mediated gene flow, which yields adventitious Bt cotton plants, and pollen-mediated gene flow, which generates outcrossed seeds. A spatially-explicit statistical analysis was used to quantify the effects of nearby Bt and non-Bt cotton fields at various spatial scales, along with the effects of pollinator abundance and adventitious Bt plants in fields, on pollen-mediated gene flow. Adventitious Bt cotton plants, resulting from seed bags and planting error, comprised over 15% of plants sampled from the edges of three seed production fields. In contrast, pollen-mediated gene flow affected less than 1% of the seed sampled from field edges. Variation in outcrossing was better explained by the area of Bt cotton fields within 750 m of the seed production fields than by the area of Bt cotton within larger or smaller spatial scales. Variation in outcrossing was also positively associated with the abundance of honey bees.

**Conclusions/Significance:**

A comparison of statistical methods showed that our spatially-explicit analysis was more powerful for understanding the effects of surrounding fields than customary models based on distance. Given the low rates of pollen-mediated gene flow observed in this study, we conclude that careful planting and screening of seeds could be more important than field spacing for limiting gene flow.

## Introduction

Gene flow between sexually compatible crops typically decreases as the distance between crops increases. Thus, growers who intend to minimize gene flow from surrounding crop varieties commonly do so by increasing the spacing between fields [1]. Nevertheless, transgene flow (i.e., gene flow of a genetically engineered trait) into commercial agricultural seed lots is documented in maize, canola, soybean, and cotton [2]–[5]. As transgenic plants, grown by 14 million farmers in 25 countries [6], are a dominant landscape feature in many regions, some transgene flow is inevitable [7], [8]. However, substantial transgene flow could threaten the intellectual property rights of biotechnology companies, markets for non-transgenic products, and resistance management strategies for insects and weeds [4], [9]–[12].

Transgene flow can occur via pollen-mediated gene flow or seed-mediated gene flow [11]. Pollen-mediated transgene flow (“outcrossing”) occurs when plants without a particular transgene are cross-pollinated by plants with the transgene. If the resulting seeds are planted, “adventitious presence” occurs in fields the following year. In contrast, seed-mediated transgene flow results from volunteer transgenic plants emerging in fields, adventitious presence in the planted seed, or human error during planting, harvesting, or seed processing. Seed-mediated gene flow can enhance pollen-mediated gene flow when “adventitious plants” arising from seed-mediated gene flow cross-pollinate surrounding plants [3], [5], [13], [14]. For cultivated cotton (*Gossypium hirsutum*, L.), which is the focus of our study, vegetative dispersal does not occur in the field [15] and, therefore, is not considered here.

Empirical field data on transgene flow are critical for modelers and decision makers who wish to develop containment strategies [1]. Most empirical studies have been relatively simple and focused on pollen-mediated gene flow [1]. While simulation models have explored the simultaneous roles of pollen vectors, field spacing, and adventitious plants on pollen-mediated gene flow rates [16], [17], statistical analyses of empirical data have not simultaneously quantified these effects. Several empirical studies have statistically described the decline in transgene flow with distance from the nearest source of transgenic plants [e.g.], [ 13], [ 18, 19], but this approach can be imprecise in complex agricultural landscapes with many sources of transgenic plants. Thus, we saw a need for a spatially explicit model that would account for the area and distance of all relevant neighboring fields, along with the effects of pollen vectors and adventitious plants, to evaluate the causes of pollen-mediated gene flow in commercial fields.

Relatively little gene flow research focuses on cotton, although it is the third most abundant genetically engineered crop [6]. This is likely because it is a self-pollinating crop with low outcrossing rates. While the ability of transgenic *Bacillus thuringiensis* (Bt) cultivars of *G. hirsutum* to cross-pollinate non-Bt *G. hirsutum* is well-documented [15], [20]–[22], pollen-mediated transgene flow rates in cotton rarely exceed 1% of seeds at a distance of 10 meters into a field [15], [20]–[23]. Nevertheless, in 2004, we found 7.5–8% adventitious presence of Bt cotton in non-Bt cotton experimental plots in Arizona, USA, likely resulting from adventitious presence in the planted seed [5]. In subsequent testing of commercial non-Bt cotton seed bags, three out of eleven bags contained 1% Bt seed, as indicated by the presence of the Bt protein Cry1Ac [5]. The source of this gene flow was unknown [5].

Outcrossing in cotton is mediated by bees and not by wind [23], which presents a challenge for modelers, because the precise relationship between pollinators and gene flow is difficult to quantify [1]. Two studies of transgene flow in cotton each reported that a location with abundant bees had higher outcrossing than a location with few bees [22], [23]. However, while knowledge of pollinator effects is crucial for modeling gene flow in insect-pollinated crops [24], other field studies have not precisely quantified the effect of pollinator density on transgene flow rates in cotton or any other crop.

Here, we evaluated the relative importance of pollen- and seed-mediated gene flow in the spread of the *cry1Ac* transgene into non-Bt cotton seed production fields, and developed a spatially-explicit statistical model for characterizing gene flow from multiple fields. We used geographic information system (GIS) and multiple logistic regression tools to simultaneously test the hypotheses that pollen-mediated gene flow would: 1) increase as the area of nearby Bt cotton fields increased, 2) decrease as nearby non-Bt cotton increased [25], 3) increase as the abundance of pollinating insects increased, and 4) increase as the abundance of adventitious Bt cotton plants increased. We also evaluated the spatial scale of pollen-mediated gene flow, the extent of seed-mediated gene flow from volunteer plants, and adventitious presence in the planted seed.

## Methods

Transgene flow from Bt cotton to non-Bt cotton was monitored in approximately 130 ha of non-Bt cotton seed production fields in Arizona, USA in 2007. Such fields are grown by farmers under contract with seed companies and are used to produce both lint and seed. We selected three farms in western, central, and eastern Arizona, respectively, that we believed to be representative of cotton seed production fields in Arizona. From these farms, 15 non-Bt cotton seed production fields, which ranged from 2.5 to 16 ha, were selected based on, 1) availability of subsampled seed from the planted seed lot, 2) receiving news of the field before the rows were cultivated for weed management, 3) accessibility, and 4) maximizing the distance between monitored fields (no adjacent fields were selected). Although we used the Bt protein Cry1Ac as a marker for gene flow from Bt cotton, we note that some cotton grown in Arizona produces two Bt proteins: Cry1Ac and Cry2Ab. Five non-Bt cotton varieties were represented in the monitored fields, of which four varieties were transgenic for glyphosate resistance.

### Examining Sources of Seed-Mediated Gene Flow

We tested seed from the six seed lots used in planting the 15 monitored fields for Cry1Ac. Seed samples were provided by growers and were collected from seed bags or recently filled hoppers on the planting equipment. When possible, we collected multiple seed samples from a seed lot for archiving. From each seed lot, 200 seeds were tested with a lateral flow immunoassay (Cry1Ab/Ac ImmunoStrips, Agdia Inc., Elkhart, IN). Each seed was halved, with one half of the kernel tested in a pool and the other half archived. Pools of 25 seed halves were tested together, with pools of 24 non-Bt seed halves plus one Bt seed half serving as positive controls, and buffer as the negative control. We followed the manufacturer's protocol, but increased extraction time from 30 s to 2 h to yield clearer test results [5]. All controls (20 positive, 20 negative) produced expected results. For pools testing positive, archived seed halves were tested with ImmunoStrips following the manufacturer's guidelines to quantify the number of Bt seeds in the pool [5]. The proportion of adventitious presence of the *cry1Ac* transgene in each seed lot was estimated as the number of Cry1Ac positive seeds divided by the total number of seeds tested.

To quantify volunteer plants emerging from the soil seed bank, we walked a minimum of four transects through each field, inspecting a minimum of eight rows soon after plants emerged but before rows were cultivated to manage weeds. We noted and sampled cotton plants outside of rows and residual cotton lint with seeds in the soil.

### Assessing Factors That Enhance Pollen-Mediated Gene Flow

We monitored pollinator activity in fields every two weeks throughout peak flowering with visual surveys. Fields were monitored two to five times, depending on their flowering period and accessibility. Fields were inaccessible during flood irrigation, and some fields were frequently flooded. For visual monitoring, an entomologist walked a consistent pace (∼0.5 m/s) along the centermost row of a field and both edge rows, counting the number of open white flowers and the number of pollinating insects (i.e., insects moving among flowers and foraging inside flowers) [26]. Thus, approximately 5,000–13,000 plants, depending on field size, were surveyed during each monitoring, which lasted 20 min. to 1 hr. For consistency, the same entomologist performed all monitoring. Honey bees (*Apis mellifera* L.) were identified to species while other pollinators were recorded and, when possible, collected for future identification. Nearly all pollinating insects were bees, with moths and wasps seen on rare occasion. Bumble bees (*Bombus spp.*) were not seen. For each field, the average number of honey bees and native bees per flower (i.e., bee densities) were separately calculated by dividing the total number of honey bees or native bees by the total number of flowers observed across monitoring dates [26].

**Figure 1:**
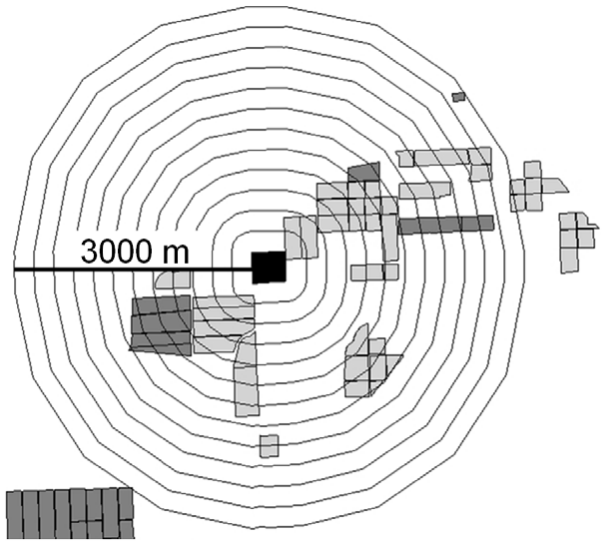
Diagram of rings drawn around a hypothetical cotton field. The first ring is 250 m from the field edge, and each subsequent ring increases in radius by 250 m. The area of non-Bt and Bt cotton was measured at each increasing scale. Light and dark gray represent non-Bt and Bt cotton, respectively, and the black rectangle represents a monitored non-Bt cotton field. For actual monitored fields, some rings overlapped those of nearby monitored fields.

Maps of all Arizona cotton fields in 2007, including identities of non-Bt and Bt cotton fields, were obtained from the Arizona Cotton Research and Protection Council [27]. Using ArcView GIS Version 3.1 [28], we drew twelve rings around the edge of each seed production field, with the first ring 250 m from the field edge, and each successive ring increasing in distance by 250 m (Fig. 1). The area of Bt and non-Bt cotton between the field edge and each ring (m^2^) was calculated with ArcView [29]. We observed substantial overlap in the flowering periods of monitored fields and neighboring Bt and non-Bt cotton fields.

### Plant Sampling and Analysis

While monitoring of pollinators and volunteer plants was performed in both edge and middle rows, we focused on sampling edge plants at the time of harvest. Pollen-mediated gene flow rates in cotton tend to be low and therefore are easiest to detect at the field edge, where rates tend to be highest [22]. Therefore, focusing on field edges allowed us to draw connections between explanatory variables and outcrossing rates by testing hundreds of seeds per field from the edges, rather than thousands of seeds from the center.

For each field, shortly before harvest, we sampled mature cotton bolls from each of 100 plants (one boll per plant) from the four outer edges of the field (25 plants per edge). We equally sampled bolls from low, middle and high positions on the plants [20]. We sampled plants from the centermost 25 m of each field edge, as defined with GPS (eTrex Legend, Garmin). We also sampled 25 plants from corresponding interior sections 20 m into the field from each edge, but bolls from some of the interior sections were not analyzed (see below).

To assess pollen- and seed-mediated gene flow, bolls were tested for Cry1Ac with ImmunoStrips. We first tested bolls from field edges. Then, for each field from which outcrossing was identified at the edge, we randomly selected one edge with outcrossing and tested bolls from its corresponding interior sample. This method allowed us to investigate outcrossing levels further into the fields. Although we only collected full-sized bolls, some bolls from edge samples did not contain mature, testable seeds, decreasing the number of replicates (Table 1). In all, we analyzed samples from 1,211 plants (12,908 seeds from 1,211 bolls) from edges and, from fields with detected outcrossing, 240 plants (2,400 seeds from 240 bolls) from the interiors (Table 1).

**Table 1:** Pollen-mediated gene flow of the *cry1Ac* transgene in non-Bt cotton fields, sample sizes, and field attributes.

Field	Plants (*n*)^1^			Distance to nearest Bt cotton field (m)	HB/100 flowers^2^	Pollen-mediated gene flow (% of seeds)		
	Total Edge	Paired Edge	20 m			Total Edge	Paired Edge	20 m
*A*	77	15	24	727	0.15	0.63	3.1	0
*B*	78	15	24	245	0.25	0.17	1.0	0
*C*	87	24	24	5	0.033	0.48	0.83	0
*D*	78	---	---	11	0	0	---	---
*E*	78	15	24	33	0	0.13	0.67	0
*F*	96	24	24	8	0.014	0.42	1.7	0
*G*	78	15	24	578	0.45	0.51	1.3	0
*H*	78	---	---	951	0.28	0	---	---
*I*	78	24	24	835	2.4	0.13	0.42	2.6
*J*	67	24	24	666	1.5	0.15	0.42	0
*K*	87	24	24	12	0.8	0.71	0.87	1.7
*L*	78	24	24	943	2.5	0.13	0.44	0.83
*M*	77	---	---	1997	2.2	0	---	---
*N*	87	---	---	9	0	0	---	---
*O*	87	---	---	9	0	0	---	---

1 Number of tested plants, including the total number of edge plants, the number of edge plants included in the paired analysis (where applicable), and the number of plants collected 20 m in from the field edge for paired analysis (where applicable).

2 Honey bee (HB) density from visual monitoring (honey bees/100 flowers).

From each tested boll, we first tested 10 subsampled seeds as a pool and followed up with individual seed tests for Cry1Ac positive pools, as described above for seed bag samples. For bolls with <10 mature seeds, all seeds were tested in the pool. We also tested tissue from the pericarp (i.e., fruit wall) of bolls with Bt seeds to differentiate between adventitious Bt plants and non-Bt plants outcrossed by Bt pollen [5]. Bt-outcrossing (pollen-mediated gene flow) was identified by bolls with Bt toxin detected in some of the seeds but not in the maternal pericarp tissue. However, adventitious Bt plants (seed-mediated gene flow) were identified by detectable Bt toxin in both seeds and pericarp tissues. Adventitious Bt plants were further sorted by whether they contained only Bt seeds or both Bt and non-Bt seeds. Bt plants producing both seed types are hemizygous and average 75% seeds with the Bt trait when they self-pollinate [30]. Calculating the relative proportions of hemizygous versus homozygous plants yields insight into the source of adventitious plants, as hemizygous plants result from cross-pollination events between Bt and non-Bt cotton in previous generations [5].

Controls were run simultaneously with ImmunoStrips tests. For seed pool tests, we used 10 pooled non-Bt cotton seeds as negative controls, and one Bt cotton seed plus nine non-Bt cotton seeds as positive controls. Seventy pairs of controls were run, and all produced expected results. For individual seed tests, 20 control pairs of individual Bt and non-Bt cotton seed halves were tested and produced expected results. For pericarp testing, pericarp samples from Bt and non-Bt cotton bolls were used as controls. Out of seven control pairs, one negative control produced a weak false positive result. As expected, all samples with positive pericarp tests contained ≥60% Bt seeds, while samples with negative pericarps had ≤20% Bt seeds, confirming the test's utility for differentiating between pollen- and seed-mediated gene flow [5].

### Statistics

We used multiple logistic regression followed by likelihood ratio tests to assess the effects of the explanatory variables on the odds of pollen-mediated gene flow. To do this, we used the nominal logistic regression platform and the generalized linear model platform in JMP 8.0 (SAS Institute [31]). Both platforms produced the same results, but the nominal logistic regression platform provided odds ratios and their confidence intervals, while the generalized linear model platform facilitated tests for overdispersion. To avoid bias, the procedure for building our statistical model was determined in advance, including the experimental unit, response variable, statistical test, and criteria for excluding explanatory variables from the final model.

Because the same bee visit could result in cross-pollination of multiple ovules in a cotton flower, we considered individual bolls, rather than individual seeds, as the experimental unit in statistical analyses. This is identical to an analysis with individual plants as the experimental unit, as only one boll was collected from each sampled plant. The response variable was a binomial count of the number of Bt-outcrossed and non-outcrossed seeds in individual bolls from non-Bt cotton plants at the edge of monitored fields. Explanatory variables included the total area of Bt cotton and the total area of non-Bt cotton in a designated ring around each monitored field, pollinator density in the monitored field (honey bees or native bees per flower), and the proportion adventitious Bt cotton plants at the edge of the monitored field. Transformations of explanatory variables were performed, as needed, to meet assumptions of linearity and homogeneity of the residuals. A summary of the explanatory variables and their transformations is included in Table 2.

**Table 2:** Summary of explanatory variables included in the full logistic regression analysis.

Variable	Transformation	Constant across scales of analysis?
1. Honey bee density (bees per flower)	arcsine√x	Yes. Measurement was from the monitored field.
2. Native bee density (bees per flower)	arcsine√x	Yes. Measurement was from the monitored field.
3. Area of Bt cotton in neighboring fields (ha)	log (x+1)	No. Variable calculated separately for each spatial scale of analysis.
4. Area of non-Bt cotton in neighboring fields (ha)	log (x+1)	No. Variable calculated separately for each spatial scale of analysis.
5. Proportion of plants that were adventitious Bt cotton plants	arcsine√x	Yes. Measurement was from the monitored field.
6. Interaction between variables 3 and 5	N/A	No. Contained variable 3, which changed with scale.

Variables that were not significant (α>0.05) at any of the spatial scales in the model with all 15 fields were excluded from further analyses.

The analysis was performed separately for each spatial scale (Fig. 1), with the area of nearby Bt and non-Bt cotton fields varying among spatial scales, while bee densities and the proportion of adventitious Bt plants remained constant. We also considered the interaction between adventitious Bt plants and the area of Bt cotton at each spatial scale, as we suspected that adventitious Bt plants would diminish the association between nearby Bt cotton fields and outcrossing, based on findings from our 2004 field study [5].

The uncertainty (U) coefficient of determination (R^2^) is the proportion of variation (uncertainty) in the dataset that is attributable to the logistic regression model. This parameter is equivalent to the R^2^ used in linear regression, but tends to be much lower in logistic regression because it depends on the negative sum of the logs of observed probabilities [31]. As we increased the spatial scale of analysis (Fig. 1), we expected R^2^ to increase if the added area helped to explain outcrossing, but to decrease once the scale exceeded the distance to which outcrossing occurred. Thus, we plotted R^2^ for each spatial scale and used the scale with a maximum R^2^ in our final analysis [29]. Explanatory variables for which *P*>0.05 at all spatial scales of the analysis were excluded from the final model.

Previous studies modeled gene flow as a function of distance from the nearest transgenic source field. To compare this method with our spatially-explicit approach, we performed a logistic regression analysis where the shortest distance from each monitored field to the nearest Bt cotton field (log transformed) was substituted for the area of neighboring Bt cotton. For both the distance model and spatially-explicit model, deviance goodness-of-fit tests and overdispersion parameters (values ≠1 conflict with the assumption of binomial distribution) were used to determine whether the sample data followed a binomial distribution, and corrections for overdispersion were applied where needed [31].

Finally, we compared outcrossing in samples from the edge of fields versus the interior of fields (20 m inside of fields) to test the hypothesis that outcrossing declines with distance into a field. To do this, for each field we subtracted the proportion of sampled bolls from non-Bt cotton plants that contained Bt-outcrossed seeds in the interior samples from the proportion in their paired edge samples. We then used a one-tailed, paired *t*-test to determine whether this difference was greater than zero. All of the above statistics were performed with JMP 8.0 [31].

To determine the sampling power of our study, we ran a resampling program where 1000 samples of the sizes used in our study were drawn from a population with a hypothesized rate of Bt seeds or plants. Averaged across samples, adventitious presence was always equal to the rate specified in simulations, but some samples did not detect Bt seeds or plants. From these simulated samples, we determined the proportion from which at least one positive seed or plant was detected. In our testing of seed lots (n = 200 seeds/lot), we had an 86.0% chance of detecting adventitious presence in a seed lot if the gene flow rate was 1%, and a 98.8% chance of detecting it if the rate was 2%. Our rate of detecting Bt-outcrossed seeds in any given cotton field (n =  ∼800 seeds/field) was 87.7% if the true outcrossing rate was 0.25%, and 98.1% if the outcrossing rate was 0.5%. The probability of detecting adventitious Bt cotton plants in an individual field at our sample size of ∼80 plants per field was 96.5%, 86.8%, or 62.3% if the true proportion of adventitious Bt cotton plants was 3.75% (3/80), 2.5% (2/80), or 1.25% (1/80), respectively.

## Results

Two of the six seed lots used to plant the monitored non-Bt cotton seed production fields contained detectable levels of Bt cotton seed, as indicated by presence of the Cry1Ac protein. Seed Lot I contained 20% Bt seed, while Seed Lot II contained 0.5% Bt seed (Table 3). After finding the seed bag with 20% Bt seed, we tested 25 seeds from a second seed bag from the same seed lot and found 28% Bt seed. Seed Lot I was used to plant two of the 15 monitored fields, from which 17% (field *A*) and 23% (field *B*) of plants sampled from field edges were adventitious Bt plants (Table 3). Thus, adventitious presence of the *cry1Ac* transgene was consistent throughout this seed lot based on two estimates from seed bags (mean  = 24%) and two estimates from tested cotton plants (mean  = 20%). Plotting the distribution of adventitious Bt plants across fields revealed fields *A* and *B* to be outlier data points. Therefore, logistic regression analyses for outcrossing were performed with and without these fields.

**Table 3:** Seed-mediated gene flow of the *cry1Ac* transgene in monitored non-Bt cotton fields.

Field	Seed lot	Adventitious presence in planted seed (%)	Adventitious plants^1^ (%)		Hemizygous^2^ (%)	Source^3^
			Edge	20 m		
*A*	I	20	17	17	5.9	Seed bag
*B*	I	20	23	25	4.2	Seed bag
*C*	II	0.5	28	0	13	Planting error
*D*	II	0.5	0	---	---	---
*E*	II	0.5	0	0	---	---
*F*	II	0.5	1.0	0	100	Seed bag
*G*	III	0	0	0	---	---
*H*	III	0	0	---	---	---
*I*	IV	0	0	4.2	100	Unknown
*J*	IV	0	0	4.2	100	Unknown
*K*	IV	0	2.3	0	100	Unknown
*L*	IV	0	1.3	0	0	Unknown
*M*	V	0	0	---	---	---
*N*	VI	0	1.1	---	0	Unknown
*O*	VI	0	2.3	---	0	Unknown

1 Percentage of plants that were adventitious Bt cotton plants in samples taken from the field edge or 20 m in from a field edge, if applicable.

2 Percentage of adventitious Bt cotton plants that were hemizygous for the Bt trait.

3 Putative source of seed-mediated gene flow.

A high estimated rate of adventitious presence in a third field was attributed to planting error (field *C*, Table 3). All plants tested from one edge were adventitious Bt plants (n = 24), yet no plants from the other three edges contained Cry1Ac (n = 63). We tested one plant from the corresponding interior sample to determine the extent of the planting mistake. It was negative, indicating that fewer than 20 rows were affected. Because adventitious presence was not uniform throughout field *C*, the misplanted edge was considered to be part of an adjacent Bt cotton field for statistical analyses. Adventitious Bt plants were identified in 10 of the 15 fields, with a median rate of 1% of plants sampled from field edges (Table 3).

Pollen-mediated gene flow from Bt cotton was rare (Table 1). On average, only 0.23% of seeds from non-Bt cotton plants at field edges contained Cry1Ac (n = 15 fields, 95% confidence interval (CI)  = 0.092–0.37%). At any scale of analysis (Fig. 1), the area of neighboring non-Bt cotton and the density of native bees in monitored fields were not significantly associated with the odds of Bt-outcrossing of non-Bt cotton plants (*P*>0.05), after accounting for the effects of the other explanatory variables. Thus, these factors were excluded from the statistical model.

**Figure 2:**
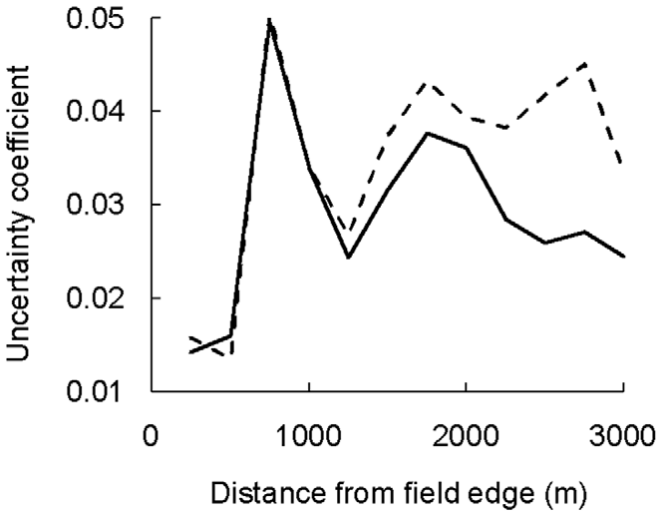
Uncertainty coefficient of determination (R^2^) for multiple logistic regression of pollen-mediated gene flow. The area of Bt cotton at various distances from the edge of monitored non-Bt cotton fields was considered in separate analyses for each scale. Honey bee density, the proportion of plants in the monitored non-Bt cotton fields that were adventitious Bt plants, and the interaction between Bt cotton fields and adventitious Bt plants were also in the analyses. Pollen-mediated gene flow of the *cry1Ac* transgene was the response variable for the analyses. Results with fields *A* and *B* (solid line) and without fields *A* and *B* (dashed line) are shown.

Our final model of pollen-mediated gene flow included the density of honey bees in monitored fields, the proportion of adventitious Bt cotton plants in monitored fields, the area of Bt cotton fields surrounding the monitored fields (using various spatial scales of analysis, Fig. 1), and the interaction between these last two factors. The uncertainty coefficient of determination (R^2^) peaked at a scale of 750 m from the field edge for models with and without fields *A* and *B* (Fig. 2). However, R^2^ was lower when scales beyond 750 m (1000–3000 m) were considered, suggesting that Bt cotton at distances of more than 750 m from the field edge did not affect outcrossing (Fig. 2). Therefore, we assessed factors affecting outcrossing at the 750 m scale.

At the 750 m scale, the area of Bt cotton surrounding a seed production field and the density of foraging honey bees were positively associated with the odds of Bt-outcrossing of non-Bt cotton plants for models with or without fields *A* and *B* (Table 4, Table 5). For the model with all 15 fields, the proportion of adventitious Bt cotton plants in the monitored fields was also positively associated with Bt-outcrossing (Table 4, Table 5), and there was a significant negative interaction between the area of nearby Bt cotton fields and adventitious Bt plants (Table 4). Thus, as the proportion of adventitious Bt plants in seed production fields increased, the effect of nearby Bt cotton fields on outcrossing rates declined. However, the contribution of adventitious Bt cotton plants was not statistically significant in the model without fields *A* and *B* (Table 4). The equation for the odds of pollen-mediated gene flow in the final model with all 15 fields was: logit(π)  =  −11.7+22.0(honey bee density) +0.40(area of Bt cotton within 750 m) +17.4(adventitious Bt plants) – 1.5(area of Bt cotton within 750 m)(adventitious Bt plants). The following very similar equation describes the model with 13 fields: logit(π)  =  −11.5+22.8(honey bee density) +0.38(area of Bt cotton within 750 m) +10.7(adventitious Bt plants) – 1.1(area of Bt cotton within 750 m)(adventitious Bt plants). See Table 2 for details on the transformations of the above explanatory variables. There was no evidence of overdispersion, as the overdispersion statistic was 1.5 and lack of fit was not significant (χ^2^ = 15, *P* = 0.14, and χ^2^ = 14, *P* = 0.072 for the models with and without fields *A* and *B,* respectively).

**Table 4:** Effect likelihood ratio tests for pollen-mediated gene flow of the *cry1Ac* transgene in monitored non-Bt cotton fields.

Explanatory variable	15 fields		13 fields	
	χ^2^	Significance	χ^2^	Significance
Honey bee density	10.4	*P* = 0.0013	10.4	*P* = 0.0013
Area of Bt cotton within 750 m^1^	15.5	*P*<0.0001	13.0	*P* = 0.0003
Adventitious Bt plants (%)	11.5	*P* = 0.0007	0.66	*P* = 0.42
Interaction	10.0	*P* = 0.0016	0.96	*P* = 0.33

Significance levels (*P*-values) for each factor from models with and without fields *A* and *B* (Table 1, Table 3) are given. See Table 2 for details on the explanatory variables.

1 Area of Bt cotton fields within 750 m of the edge of monitored non-Bt cotton fields.

**Table 5:** Range odds ratios^1^ for the effects of the explanatory variables on outcrossing.

Explanatory variable	15 fields		13 fields	
	Odds ratio^1^	Confidence interval	Odds ratio^1^	Confidence interval
Honey bee density	6.4	1.1–39	30	3.7–270
Area of Bt cotton within 750 m	9.1	1.5–65	84	6.3–2900
Adventitious Bt plants (%)	2.3	0.63–6.9	---	---

From a simplified model without the interaction term (odds ratios of interactions are difficult to interpret). Results from models with and without fields *A* and *B* are given.

1 Range odds ratios estimate the change in the odds of an event (i.e., outcrossing) over the observed range of an explanatory variable [31]. For instance, in the field with the most honey bees, plants had 6.4-fold higher odds of outcrossing than in the field with the fewest honey bees for the 15 field model.

The distance between the monitored fields and their nearest neighboring Bt cotton fields (log transformed) was negatively correlated with the area of Bt cotton within 750 m of the monitored fields (log transformed) (r =  −0.86, *P*<0.0001). For the analysis based on distance, lack of fit was significant (χ^2^ = 28, *P* = 0.0017, and χ^2^ = 24, *P* = 0.0022 for models with and without fields *A* and *B*, respectively; overdispersion  = 1.9 and 2.1, respectively). Because lack of fit was significant, we corrected for overdispersion in the distance model [31]. With or without fields *A* and *B*, after correcting for overdispersion, there was no significant association between outcrossing and distance to the nearest Bt cotton field (*P*≥0.12), or the other factors in the model, including honey bee density (*P*≥0.12), adventitious Bt plants (*P*≥0.11), and the interaction between distance and adventitious Bt plants (*P*≥0.28).

In the experiment comparing paired edge and interior field samples, there was a trend for a decline in the proportion of non-Bt cotton bolls containing Cry1Ac positive seeds from the edge to the interior of fields (Table 1), but this trend was not statistically significant (*t*_9_ = 1.6, one-sided *P* = 0.072). The presence of adventitious Bt plants (i.e., seed-mediated gene flow) did not differ between edge and interior samples either (paired *t*-test excluding the planting mistake in field *C*, *t*_9_ = 0.39, two-sided *P* = 0.71). Similarly, honey bee densities appeared consistent across fields, with no difference between edge and middle rows (paired *t*-test, *t*_14_ = 1.1, two-sided *P* = 0.29). Honey bees comprised 88% of the observed foraging bees, while native bees were less abundant in all fields (<0.5 native bees per 100 flowers).

The seed composition of bolls revealed that ten of the 74 identified adventitious Bt plants (13.5%) were hemizygous for the *cry1Ac* transgene (see Table 3). Bolls from these plants contained, on average, 79% (95% CI  = 72-86%) Bt seeds, which is not significantly different from the 3∶1 ratio for hemizygous cotton plants that self-pollinate (*t*_9_ = 1.3, *P* = 0.24).

We found no evidence that volunteer plants contributed to gene flow. Fewer than two plants per kilometer of monitored row (<0.01% of plants) emerged outside of planted rows, even in fields where residual cotton lint was visible. Moreover, rare plants outside of rows could have resulted from flaws in the planting machinery. As volunteer plants were an unlikely source of gene flow, we did not follow up with ImmunoStrips tests of the plants occurring outside of rows.

## Discussion

Although seed-mediated gene flow has received less attention than pollen-mediated gene flow in the literature [1], it was clearly the most prominent source of *cry1Ac* transgene flow in this study (Table 1, Table 3). Seed-mediated gene flow resulted primarily from adventitious presence in the planted seed and from planting error, although some fields with no evidence of these sources contained low percentages of adventitious Bt plants (Table 3). Some adventitious Bt plants were hemizygous for *cry1Ac* (Table 3), indicating pollen-mediated gene flow in previous generations, either of Bt pollen into non-Bt cotton plants, or of non-Bt pollen into adventitious Bt cotton plants [5]. In fields where gene flow entered via the planted seed, most adventitious Bt plants were homozygous, suggesting that seed-mediated gene flow was the original source of gene flow (Table 3, fields *A–F*).

Pollen-mediated gene flow of the *cry1Ac* transgene was also observed, but occurred at rates below 1% at field edges (Table 1). While other authors have noted the relevance of pollinator abundance, adventitious plants, and the area of surrounding crops to pollen-mediated gene flow [1], [16], [17], to our knowledge, this is the first empirical study to statistically describe the concurrent effects of these factors on gene flow rates. We showed that a spatially-explicit analysis based on the area of nearby crops compared favorably to the simplest distance-based analysis. Honey bees appeared to be the primary outcrossing agent in the seed production fields, which was also noted in previous cotton outcrossing studies [e.g.], [ 22], [ 26, 32]. Native bees did not appear to increase outcrossing significantly, perhaps due to their low abundance. The area of Bt cotton fields within 750 m of the monitored fields best explained outcrossing rates, as the explanatory power of the model was lower at smaller or larger scales (Fig. 2). The 750 m scale of outcrossing falls within the foraging range of honey bees, which has been documented at over 3000 m [33]. We expected neighboring non-Bt cotton fields to reduce Bt-outcrossing by acting as an alternative sink for Bt pollen and as a competing pollen source, but did not observe this effect at the sample size used.

We did not detect a significant difference in outcrossing between field edges and samples taken 20 m from the edge. We note that our study design could have potentially overestimated differences in outcrossing between the edge and interior samples, because we only tested interior samples if outcrossing was already detected at the corresponding edge. However, this would not affect our conclusion that no significant difference was observed at the sample size used. Similarly, in our 2004 study conducted in non-Bt cotton plots with 7.5–8% adventitious presence of Bt plants, we observed no significant decline in outcrossing with distance from the adjacent Bt cotton plots [5]. Small-scale field trials in other regions reported dramatic decreases in Bt-outcrossing with distances of 20 m or less into non-Bt cotton buffers surrounding Bt cotton test plots [15], [20], [21]. Unharvested buffers of non-transgenic plants are commonly used as a sink to contain transgenic pollen [1]. Our study showed that gene flow rates did not always drop off at 20 m. However, this result does not imply that outcrossing at the edge of fields is representative of the entire field, as samples beyond 20 m from the field edge were not taken.

We expect that pollen-mediated gene flow rates would be lower in the center of fields [22]. However, edge sampling was the most efficient way to maximize detection of outcrossing in this study, as cotton is a low outcrossing crop. We assume that the significant association between our explanatory variables and pollen-mediated gene flow rates extend to whole fields, as field edges are a point of entry into the rest of the field. A more extensive survey with higher sample sizes to detect low gene flow rates in the interior of fields would be needed to demonstrate that these explanatory variables are associated with pollen-mediated gene flow rates throughout the field.

Adventitious Bt cotton plants may have acted as a source of pollen-mediated gene flow [5], [13], [14] (Table 4), and enhanced outcrossing levels 20 m inside fields where little outcrossing from neighboring fields was expected. There was some evidence that adventitious Bt cotton plants diminished the association between neighboring Bt cotton fields and pollen-mediated gene flow (Table 4), suggesting that the two pollen sources may compete to outcross non-Bt cotton plants. However, the contribution of adventitious Bt cotton plants and the interaction between the two Bt pollen sources were only significant when fields *A* and *B*, which had high adventitious presence throughout (Table 3), were included in the analysis. Data from more fields with intermediate to high adventitious presence (i.e., 3-28%; see Table 3) would be needed to more fully detail the contributions of adventitious Bt cotton plants to outcrossing.

Other factors, in addition to those measured in this study, may also influence gene flow patterns. For example, the extent of overlap in flowering periods and characteristics of specific crop varieties may influence the extent of cross-pollination between any two crop patches [1]. The robust statistical association between the variables in our model (Table 4) suggests that pollinator abundance and the area of surrounding Bt cotton fields are key variables that influence pollen-mediated gene flow.

In the United States, non-transgenic crops do not require separation from transgenic crops that have received government approval [23], unless they are labeled as “GE-free” or “organic” [12]. The seed examined in this study did not have these labels. Furthermore, most of the non-Bt cotton varieties included in this study were transgenic for herbicide resistance, and thus were not intended for the GE-free or organic markets. Nevertheless, adventitious presence of the Bt trait is a concern for non-Bt cotton refuges used in pest resistance management programs in many countries [10], [34], [35]. Refuges are intended to increase the proportion of Bt-susceptible insects in a pest population [36]. Adventitious presence of Bt cotton in refuges could accelerate resistance by increasing the mortality of susceptible insects or shifting the dominance of resistance [10], [34].

We note that the seed produced in monitored fields may not have been sold to growers. While the United States does not have strict labeling thresholds for adventitious presence of the Bt trait in seed, seed companies sometimes voluntarily reject seed lots with adventitious presence of transgenes. However, as we observed in fields planted with the seed lot containing 20% adventitious presence (Table 3), gene flow can go overlooked and persist across generations in the seed production setting.

Although one field season does not capture variability among years, it provides a detailed snapshot of the factors that contribute to transgene flow. Results from this study suggest that crop spacing can be used to limit unwanted gene flow, as Bt cotton fields >750 m from the edge of monitored fields did not appear to contribute to outcrossing. However, pollen-mediated transgene flow rates were always low in this study (i.e., <1% of seeds at the field edge), even in monitored fields that were near Bt cotton fields (Table 1). This suggests that spacing fields hundreds of meters from transgenic crops is unnecessary for cotton, even in the European Union where the labeling threshold for adventitious presence in crops is 0.9% [37]. However, this study demonstrates the potential for seed-mediated gene flow to become prominent in settings where actions are not taken to keep adventitious presence in check. The ecological patterns underlying gene flow in this study could apply to related seed production systems, particularly for other insect-pollinated transgenic crops. In settings where seed purity is desirable, seed producers and decision makers should consider 1) screening seeds to monitor adventitious presence in the seed supply, and 2) communicating the importance of segregating seed types at planting to reduce human error.
